# Local versus intravenous injections of skeletal muscle precursor cells in nonhuman primates with acute or chronic intrinsic urinary sphincter deficiency

**DOI:** 10.1186/s13287-016-0411-3

**Published:** 2016-10-07

**Authors:** J. Koudy Williams, Gopal Badlani, Ashley Dean, Shannon Lankford, Kimberly Poppante, Tracy Criswell, Karl-Erik Andersson

**Affiliations:** 1 Wake Forest Institute for Regenerative Medicine, Wake Forest Baptist Medical Center, Wake Forest University, 391 Technology Way, Winston-Salem, NC 27101 USA; 2 Department of Urology, Wake Forest University Baptist Medical Center, Wake Forest University, 391 Technology Way, Winston-Salem, NC 27101 USA; 3 Department of Obstetrics and Gynecology, Institute for Clinical Sciences, Aarhus University, Aarhus, Denmark

**Keywords:** Intrinsic urinary sphincter deficiency, Skeletal muscle precursor cells, Stress urinary incontinence, Maximal urethral pressure, Vascularization, Somatic innervation

## Abstract

**Background:**

Many factors may influence the efficacy of cell therapy for intrinsic urinary sphincter deficiency (ISD), including the route of administration of the cells and the condition of the sphincter. The goal of this study was to compare local versus intravenous administration of autologous skeletal muscle precursor cells (skMPCs) when administered to nonhuman primates (NHPs) with either acute or chronic ISD.

**Methods:**

Thirty-two adult female monkeys were divided into eight groups (n = 4/group): (1) control; (2) surgically induced ISD/no treatment; (3) acute ISD (6-week duration)/local vehicle only; (4) acute ISD/local skMPC injection; (5) acute ISD/systemic skMPC; (6) chronic ISD (6-month duration)/local vehicle; (7) chronic ISD/local skMPC; (8) chronic ISD/systemic skMPC. Maximal urethral pressures (MUP) were measured prior to ISD, prior to treatment and at 3 and 6 months following treatment. Quantitative histology was used to measure muscle/collagen content, somatic innervation, and vascularity of the sphincter complexes.

**Results:**

In NHPs with acute ISD both systemic and local administration of skMPCs increased resting MUP values and sphincter muscle content (*p* < 0.05 vs. ISD/vehicle). However, the effects of systemic skMPC administration were significantly lower than those of local injection (*p* > 0.05). In NHPs with chronic ISD local skMPC administration had reduced (compared to NHPs with acute ISD) effects on MUP and sphincter muscle values (*p* < 0.05 vs. acute ISD/skMPC); systemic administration had no effect. Pudendal nerve-stimulated increases in MUP were significant only in acute ISD NHPs with local skMPC treatment (*p* < 0.05 vs. resting MUP). The extent of sphincter vascularization and innervation were directly related to MUP and sphincter muscle content.

**Conclusions:**

Both the chronicity of ISD and the route of cell injection influence the efficacy of cell therapy in monkey models of ISD. This may be related to the relative ability of cells to stimulate vascularization and re-innervation in these different treatment conditions.

## Background

Intrinsic urinary sphincter deficiency (ISD) is a common cause of stress urinary incontinence (SUI) and remains a significant quality-of-life issue. It is a chronic condition resulting from aging and childbirth injury to the urinary sphincter musculature and innervation, becoming clinically evident in the peri/postmenopausal years [1, 2]. Although good results of surgical therapy of SUI have been reported [3], complications are not infrequent [4] and alternative treatments may be desirable, particularly when surgical treatment has failed or if surgery poses too great a risk. Studies using adult stem cells (MSCs) to induce tissue regeneration and repair of the damaged urethral sphincter have shown positive results both in animals [5, 6] and humans [7, 8]. However, cell therapy for ISD has so far been moderately successful with consistent 50 % beneficial effects in around 50 % of women. In contrast, results of preclinical cell studies have been more optimistic [6]. However, preclinical studies most often test the effects of cell therapies on acute ISD, which does not mirror the clinical disease [1, 2]. Therefore, it seems reasonable that, to better target and predict efficacy paradigms in patients, preclinical therapeutic approaches should be tested in animal models of chronic ISD.

The route of administration of cells is clinically relevant since intravenous injection would be cheaper, less invasive, easier to administer and less painful. Intravenous administration of bone marrow progenitor cells has been explored clinically for regeneration of the myocardium and shown to be equally efficacious as direct myocardial injection [9]. In a rat model of vaginal distention-induced sphincter injury Cruz et al. reported favorable effects of intravenous administration, but this was in an acute model of ISD [10]. Whether intravenous injection is effective for ISD in the chronic setting has not been explored.

To address these issues, we tested in a nonhuman primate (NHP) model the efficacy of autologous skeletal muscle precursor cells (skMPCs) in improving sphincter structure and function, comparing routes of administration (local injection of cells versus intravenous administration of cells), and timing of treatment (acute versus chronic ISD).

## Methods

### Animal model

Studies in these adult female nonhuman primates were approved by the Wake Forest University Institutional Animal Care and Use Committee (IACUC), and were performed in compliance with the Animal Welfare Act and the Guide for the Care and Use of Laboratory Animals. Euthanasia was performed according to the standards of the American Veterinary Medical Association.

### Design

Thirty-two adult female cynomolgus monkeys (age range 8–13 years) were used in this study. Monkeys were divided into eight groups (n = 4/group): (1) control; (2) surgically induced ISD/no treatment; (3) acute ISD (6-week duration)/local vehicle only; (4) acute ISD/local skMPC injection; (5) acute ISD/systemic skMPC; (6) chronic ISD (6-month duration)/local vehicle; (7) chronic ISD/local skMPC; (8) chronic ISD/systemic skMPC. Maximal urethral pressures (MUP) were measured prior to ISD, prior to treatment, and at 3 and 6 months following treatment. Urinary sphincters were analyzed by quantitative histomorphometry 6 months posttreatment for muscle and collagen content as well as vascularization and innervation.

### The NHP model of ISD

The ISD procedure was done after baseline urodynamic measures were obtained. The monkeys were sedated with ketamine 10–15 mg/kg/intramuscularly and 1–5 % isoflurane used for induction and maintenance of anesthesia. Monkeys were prepared for aseptic surgery, anesthetized and a lower midline abdominal incision (4 cm in length) made to expose the pelvic area of the abdomen. The distal urinary tract was approached using gentle dissection of connective tissue just ventral to the urinary bladder extending dorsally to the bladder neck and caudally 2 cm to either side of the rhabdosphincter. The pudendal nerve branches supplying the sphincter (usually three in number) were identified and then selectively electrocauterized - while not damaging the sphincter directly - and then transected [11, 12]. Special care was taken not to damage surrounding structures. The abdomen was closed in two layers and postoperative support given.

### skMPC isolation and injection

A 1 cm^3^ sample of quadriceps muscle was aseptically removed from anesthetized NHPs and transported in a wash solution of Dulbecco’s phosphate-buffered saline (DPBS, HyClone, South Logan, UT, USA) with 1 % antibiotic/antimitotic (HyClone). The tissue was washed 10 min × 3 in fresh wash solution with periodic gentle agitation with a final rinse in DBPS. The sample was trimmed of unwanted tissue, weighed, and minced into fragments approximately 0.5 mm^2^ or less. Digestion media consisting of 2:1 dispase II (Sigma-Aldrich St. Louis, MO, USA): collagenase type I (Worthington, Lakewood, NJ, USA) per milliliter of basal media (custom-designed muscle progenitor cell media, PeproTech, Rocky Hill, NJ, USA) was added to the minced tissue at 1 ml per 100 mg of tissue. The sample was incubated at 37 °C, 5 % CO_2_ for 45 minutes. Upon completion, the digestion was terminated using 2 × volume of growth media [PeproTech basal media plus fetal bovine serum (FBS) and custom growth supplements] to digestion media and rigorous pipetting was applied. The suspension was filtered through a 100-micron filter and centrifuged for 5 min at 1500 RPM. The supernatant was aspirated, fresh growth media was added, and spun for a second time. Then the sample was plated on a pretreated collagen I 100-mm culture plate (BD Biocoat, Becton Dickinson, Franklin Lakes, NJ, USA) and incubated for 24 hours at 37 °C, 5 % CO_2_. The following day, the aspirate was collected and replated on a new pretreated collagen-coated plate to reduce fibroblast in the cell culture. The skMPCs were isolated and characterized as described previously [11]. Eight weeks following collection of the sample, 5 million skMPCs were suspended in 2 milliliters of Dulbecco’s modified Eagle’s medium (DMEM) without serum and injected directly into the urinary sphincter complex [at the level of the sphincter skeletal muscle layer and at four locations (12, 3, 6, and 9 o’clock positions)] of anesthetized monkeys as described previously [11, 12]. For systemic injections, the skMPCs were collected and processed identically as described above. At their respective treatment times, 5 million skMPCs were suspended in 2 ml of DMEM and injected into the saphenous vein over 30 seconds.

### Sphincter function

Monkeys were sedated and anesthetized and urodynamic measures recorded at baseline (before the nerve injury), and then prior to injections, at 3 months and 6 months postinjection using the Life-Tech Urolab Opus System V (Life-Tech Inc., Stafford, TX, USA) in combination with a 6 French Millar Mikro-Tip transducer catheter (Millar Instruments Inc., Houston, TX, USA) and a rectal balloon catheter. The catheter was inserted transurethrally and the residual urine evacuated. Urethral pressure profilometry was performed by automatic withdrawing of the sensor catheter at 0.5 mm/s. Rectal pressure was measured with a balloon catheter attached to a transducer (Life-Tech Inc., Stafford, TX, USA). Using the pressure sensors at its tip (direction upward), a static urethral pressure profile was recorded on the urodynamic machine and the maximal urethral pressure (MUP) in the region of external sphincter recorded [11, 12]. This process was repeated three times and the mean of the urethral pressure measurements calculated for each animal. MUP was then measured during pudendal nerve stimulation. The pudendal nerve stimulation site is proximal to the nerve transection and lateral to the rhabdosphincter. A Cadwell ball probe was connected to the nerve-stimulating machine (Cadwell Sierra Wave, Cadwell Laboratories, Inc., Kennewick, WA, USA) with a setting of 1 ms delay and 1 ms pulse width at 15 mA intensity. The probe was used to stimulate each nerve by direct contact. The urethral catheter was withdrawn automatically, monitoring the changes in urethral pressure. Three measures of MUP were recorded for the pudendal nerve stimulation and calculated as described for the resting MUP measures.

### Collection and analysis of tissues

The monkeys were euthanized for tissue retrieval using sodium pentobarbital (80–100 mg/kg/intravenously). The urethra, approximately 1 cm in length, was removed from the animal and immersion-fixed in 2 % phosphate-buffered formalin for 24 hours and then transferred to a 50 % sucrose solution for an additional 24 hours. Tissues were then placed in Tissue-Tek OCT Compound (Sakura Finetek, Torrance, CA, USA) and frozen in liquid nitrogen. The urethra was cut into 100 5-μm-thick cross sections. Sections used to quantify sphincter collagen and muscle content, spaced evenly along the length of the urethra, were fixed and stained with Masson’s Trichrome, which stains collagen fibers blue and muscle fibers red. Image analysis was done using the Image-Pro AMS 6.0 software (Media Cybernetics, Bethesda, MD, USA).

All immunohistochemistry was done in triplicate with a serum isotype control matched with each antibody. The antibodies used were against: neurofilament (1:200, NF 200, Sigma-Aldrich N4142); bungarotoxin (1:100, Alexa Fluor 488, B13422); and von Willebrand factor (vWF) (1:200, Dako, Carpentaria, CA, USA, A0082). To analyze the number of blood vessels, or neurofilament bundles, slides from the middle third of the sphincter complex were stained for vWF, or the neurofilament antibodies. Pictures were taken with a Leica DM4000B compound microscope (Leica Microsystems, Wetzlar, Germany) with a Retiga 2000RV Q-Imaging camera (Q-Imaging, Surrey, BC, Canada). Sections were blocked using serum-free blocking agent (Dako) to avoid nonspecific binding of primary antibodies. Primary antibody was added on the sections and incubated for 12 hours. Protein expression was identified using Texas Red anti-rabbit (Vector Laboratories Inc., Burlingame, CA, USA) The numbers of blood vessels, or neurofilament bundles were counted in five random × 100 magnification fields per section. The average number of blood vessels of each section was calculated and used to determine the group average as we have reported previously [13].

### Statistical analyses

MUP and collagen/muscle data were first analyzed with a one-way ANOVA to detect differences among groups. Logarithmic transformation was used if data were not distributed normally around the mean. If the ANOVA was significant, post hoc analysis (between groups) was performed using unpaired Student’s *t* test with a Holm-Sidak correction for multiple groups. *p* < 0.05 was considered statistically significant. Data are presented as mean plus/minus standard error of the mean.

## Results

### Sphincter collagen/muscle content

**Fig. 1:**
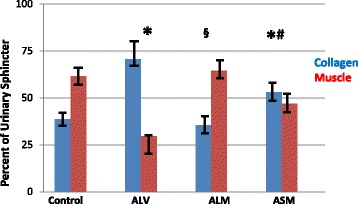
Muscle/collagen content in acute ISD. Percent of sphincter area occupied by muscle or collagen from cross sections of tissue stained with Mason’s Trichrome in monkeys with acute ISD (6-week duration). Data are shown for control (no surgery, no treatment), monkeys with acute ISD and local injection of vehicle only (ALV), acute ISD monkeys with local injection of 5 million autologous skeletal muscle precursor cells (ALM) and acute ISD monkeys with systemic administration of 5 million autologous skeletal muscle cells (ASM). ANOVA = 0.0001. Note. Statistical significance notations apply to both collagen and muscle content. * = *p* < 0.05 (for both collagen and muscle content) vs. control; § = *p* < 0.05 vs. ALV (local vehicle); # = *p* < 0.05 vs. ALM (local muscle). Values are mean ± standard error of the mean (SEM). Results indicate that ISD produced a collagen-rich sphincter, whereas local muscle injection, and to a lesser extent, systemic administration of skeletal muscle precursor cells, restored the muscle-dominant sphincter

**Fig. 2:**
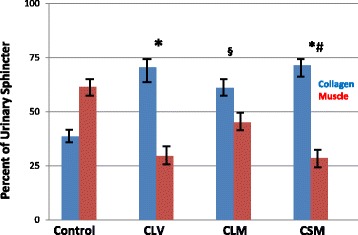
Muscle/collagen content in chronic ISD. Sphincter collagen and muscle content in monkeys with chronic ISD (6-month duration). The study groups are: control, chronic local vehicle (CLV), chronic local skeletal muscle progenitor cell injection (CLM), and chronic systemic muscle cell injection (CSM). Note. Statistical significance notations apply to both collagen and muscle content ANOVA = 0.001 * = *p* < 0.05 vs. control; § = *p* < 0.05 vs. CLV (local vehicle); # = *p* < 0.05 vs. CLM (local muscle). Values are mean ± standard error of the mean (SEM). Results indicate that the ISD procedure produced a collagen-dominant urinary sphincter and that muscle content was somewhat restored with local injection but not with systemic administration of skeletal muscle precursor cells

Figures 1 and 2 depict post hoc analysis of the muscle/collagen percentages in the urinary sphincter complex of the monkeys in the experimental groups. The data were distributed normally, thus not transformed. The ANOVA analysis was significant at *p* = 0.0001. Figure 1 presents the data from the monkeys with acute ISD. Animals treated with only local vehicle injection (ALV) produced a collagen-rich sphincter (*p* < 0.05 vs. control). Local injection of autologous skMPCs restored the collagen/muscle ratio to control values (*p* < 0.05 vs. ALV – vehicle). Systemic injection of skMPCs (ASM) had an intermediate effect in that muscle content was greater than that in vehicle only (ALV) (*p* < 0.05), but also different (less) than local skMPC (ALM) (*p* < 0.05).

Figure 2 presents the data from the monkeys with chronic ISD. Similar to the acute ISD groups, the muscle content was reduced with the ISD procedure (CLV) vs. control (*p* < 0.05). Local injection of skMPCs (CLM) increased the muscle and reduced the collagen content compared to vehicle only (CLV, *p* < 0.05), but did not reverse the collagen/muscle ratio to a muscle-dominant sphincter as it did in acute ISD. Systemic skMPC injection (CSM) had no effect on the sphincter collagen/muscle content (*p* > 0.05 vs. control and *p* < 0.05 vs. CLM).

### Resting maximal urethral pressures (MUPs)

**Fig. 3:**
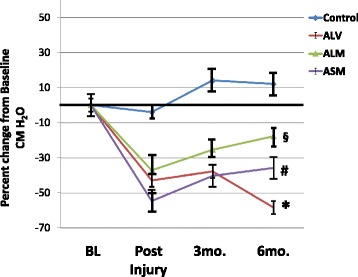
Resting maximal urethral pressures (MUP) in monkeys with acute ISD. Results are shown as the percent change from baseline values in each experimental condition. Results are shown for controls (no ISD, no treatment); monkeys with ISD and local vehicle only (ALV); monkeys with ISD and local skeletal muscle precursor cell injections (ALM) and ISD monkeys with systemic muscle cell injections (ASM). ANOVA = 0.01. * = *p* < 0.05 vs. baseline at 3 and 6 months. § = *p* < 0.05 vs. ALV. # = *p* < 0.05 vs. ALM, and *p* < 0.05 vs. ALV. Values are mean ± standard error of the mean (SEM). Results indicate that the ISD procedure reduced MUP values in all groups, and that local injection of skeletal muscle precursor cells was more effective than systemic administration in restoring baseline MUP values

**Fig. 4:**
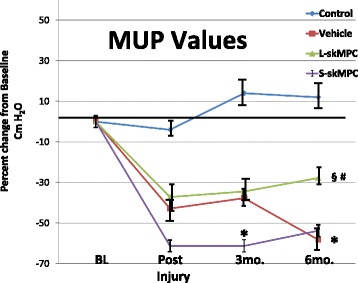
Resting MUP in monkeys with chronic ISD. Data are given as percent change in MUP from baseline values. The experimental groups are controls, chronic local vehicle (CLV), chronic local skeletal muscle precursor cell injection (CLM), or chronic systemic muscle cell injection (CSM). ANOVA *p* = 0.01; * = *p* < 0.05 vs. baseline and control at 6 months; § = *p* < 0.05 vs. baseline and control; # = *p* < 0.05 vs. CLV. All values are mean ± standard error of the mean (SEM). Results indicate that local (CLM), but not systemic (CSM) muscle cell treatment increased MUP values. However, cell therapy in chronic ISD was not as effective as in acute ISD (*p* < 0.05). *MUP* maximal urethral pressure, *skMPC* skeletal muscle precursor cells

Figures 3 and 4 depict the post hoc analysis of the resting MUPs of the monkeys in the treatment groups. The data represent logarithmic-transformed data as the data were not normally distributed (skewed). The ANOVA analysis was significant at *p* = 0.01. Figure 3 shows the data from the monkeys with acute ISD. The ISD procedure reduced maximal MUP in all groups (*p* < 0.05 vs. baseline). Injection of the carrier solution (ALV) had no effects of MUP values (*p* > 0.05 vs. injury controls = IC). Local skMPC injection (ALM) increased MUP to be similar to that of the control group (*p* > 0.05). Systemic skMPC injection (ASM) increased resting MUP values compared to AVL (*p* < 0.05), but remained lower than controls (*p* < 0.05).

Figure 4 depicts the resting MUP data from the monkeys with chronic ISD. Similar to monkeys with acute ISD, the ISD procedure markedly reduced the resting MUP values (*p* < 0.05 vs. baseline). Local (CLM), but not systemic (CSM) skMPC injections (CLM), increased resting MUPs values compared to vehicle injection only (*p* < 0.05), but not to the extent as with acute ISD (*p* < 0.05) and remained lower than controls (*p* < 0.05).

### Pudendal nerve-stimulated MUPs

**Fig. 5:**
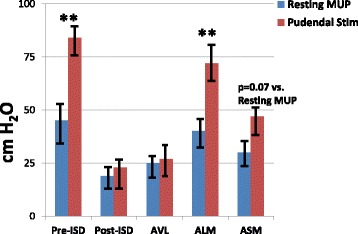
Pudendal nerve-stimulated MUP in acute ISD. Data are MUP values in cm H_2_O. The time points are pre-ISD procedure (baseline), post-ISD (immediately before treatment - 6 weeks post ISD procedure) and 6 months posttreatments in the local vehicle injection (ALV), local skeletal muscle precursor cell (ALM), and systemic muscle cell injection (ASM) groups. ANOVA *p* = 0.03. ** = *p* < 0.01 resting vs. pudendal nerve-stimulated MUP values. Values are mean ± standard error of the mean (SEM). Results indicate that pudendal stimulation increased MUP values in the local cell injection group, and that there were trends (not statistically significant) toward increasing MUP values in the systemic cell-treated group. *ISD* intrinsic urinary sphincter deficiency, *MUP* maximal urethral pressure

**Fig. 6:**
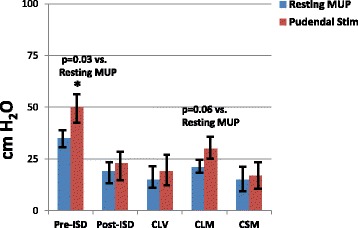
Pudendal nerve-stimulated MUP in chronic ISD. Data are MUP values in cm H_2_O. The time points are pre-ISD procedure (baseline), post-ISD (immediately before treatment - 6 months post ISD procedure) and 6 months posttreatments in the local vehicle injection (CLV), local skeletal muscle precursor cell (CLM) and systemic muscle cell injection (CSM) groups. Values are mean ± standard error of the mean (SEM). ANOVA *p* = 0.03. * = *p* < 0.05 resting vs. pudendal nerve-stimulated MUP values. Results indicate that the ISD procedure reduced the MUP response to pudendal nerve stimulation and that local skeletal muscle precursor cell injections increased MUP values, but only to the *p* = 0.06 confidence level. *ISD* intrinsic urinary sphincter deficiency, *MUP* maximal urethral pressure

Figures 5 and 6 depict the results of resting vs. pudendal nerve stimulation-induced MUPs in the monkeys with acute vs. chronic ISD. MUP was measured before and after stimulation. The difference in MUP values between the monkeys with acute or chronic ISD was measured. To normalize the data, a logarithmic transformation was done. The ANOVA analysis was significant as *p* = 0.003.

Figure 5 presents the results of the monkeys with acute ISD. The ISD procedure diminished (*p* > 0.05 vs. resting MUP) and local skMPC injections (ALM) restored (*p* < 0.05 vs. resting MUP) MUP during pudendal nerve stimulation. Injection of systemic skMPCs (ASM) restored some of the pudendal nerve responsiveness, but results were not statistically significant (*p* = 0.07).

The responses in the monkeys with chronic ISD are shown in Fig. 6. The ISD procedure diminished the pudendal nerve-stimulated increases in MUP compared to control (*p* < 0.05). Local skMPC treatment restored some of the pudendal nerve responsiveness, but results were not statistically significant (*p* = 0.06).

### Vascularization and somatic innervation

**Fig. 7:**
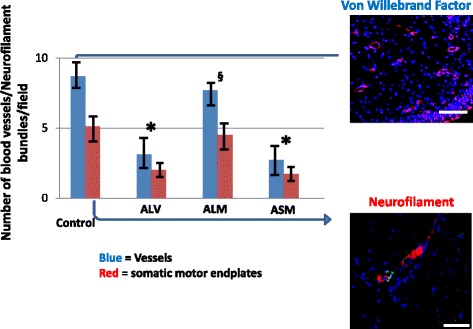
Vascularization and motor endplate expression in acute ISD. Data are the number of blood vessels using quantitative immunohistochemical evaluation of vascularization (number of von Willebrand-stained vessels) and number of somatic innervation (number of neurofilament + bungarotoxin-stained motor endplates). The study groups are control (no ISD, no treatment), local vehicle (ALV), local skeletal muscle precursor cell injection (ALM) and systemic muscle cell injection (ASM). ANOVA = 0.03. Values are mean ± SEM. * = *p* < 0.05 vs. control for both vessels and somatic nerve. § = *p* < 0.05 vs. ALV for both von Willebrand and neurofilament/bungarotoxin staining. Results indicate that the ISD procedure reduced the vascularization and somatic nerve protein expression in the urinary sphincters and that local, but not systemic, cell injections restored the expression to that seen in control animals

**Fig. 8:**
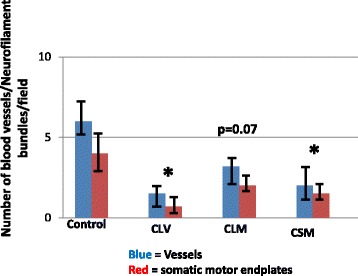
Vascularization and motor endplate expression in chronic ISD. Data are the number of blood vessels using quantitative immunohistochemical evaluation of vascularization (number of von Willebrand-stained vessels) and number of somatic innervation (number of neurofilament + bungarotoxin-stained motor endplates). The study groups are control (no ISD, no treatment), local vehicle (CLV), local skeletal muscle precursor cell injection (CLM) and systemic muscle cell injection (CSM). ANOVA = 0.03. Values are mean ± SEM. * = *p* < 0.05 vs. control for both von Willebrand and neurofilament/bungarotoxin staining. Results indicate that the ISD procedure reduced the vascularization and somatic nerve protein expression in the urinary sphincters and that local cell injections only partially restored the expression to that seen in control animals (*p* = 0.07)

Figures 7 and 8 depict the relative amount of vascularization (von Willebrand factor) and somatic innervation (neurofilament) in the urinary sphincters of the treatment groups. ANOVA = 0.03. To confirm motor endplates, the neurofilament bundles were co-stained with bungarotoxin (figures shown to the right of the graph). Figure 7 presents the data for vessel and somatic nerve bundles for monkeys in the acute ISD treatment groups. The patterns were similar as those presented for MUP and sphincter muscle. The ISD procedure reduced vessel and somatic bundle counts (*p* < 0.05 vs. control). Local skMPC treatment resulted in significantly increased number of both vessels and somatic bundles (*p* < 0.05 vs. vehicle only). In general, the amount of vascularization and neurofilament bundles was lower in the monkeys with chronic ISD compared to acute ISD (Fig. 7 vs. Fig. 8) (*p* < 0.05). Local cell injections produced some increase in sphincter vascularization and somatic motor endplate protein expression in monkeys with chronic ISD (Fig. 8), but only at the 0.07 confidence level.

## Discussion

The major findings of this study were that: (1) local injection of skMPCs was more effective than intravenous administration of the cells with respect to restoring urinary sphincter structure and function in both acute and chronic ISD; (2) local injection of skMPCs was less effective in restoring sphincter structure and function in chronic than in acute ISD; and (3) treatment effects on changes in sphincter structure and function generally mirrored changes in sphincter vascularization, somatic innervation, and pudendal nerve-stimulated increases in maximal urethral pressures.

Cell therapies for the treatment of human SUI have shown varying effects, and there is no apparent consensus on how studies should be designed, what cells should be injected, and how effects should be evaluated. Few preclinical studies simulate chronic SUI in patients. However, there seems to be a general agreement that to accelerate clinical progress more predictive preclinical research should be performed. This, in turn focuses interest on what predictive animal models and cell types/fractions should be used.

Many animal models of SUI have been described and discussed in detail [5, 6, 14–16]. Each has its benefits in further defining potential treatments and identifying underlying mechanisms of SUI. However, to mimic a clinical situation, the model should reflect a stable chronic state before intervention, i.e., a condition where spontaneous recovery is not expected. If the intervention shows improvement under these conditions, the results may have a more predictive clinical impact. Despite some differences in the encircling striated muscle in the urethra [17], the outflow region of nonhuman primates is the closest to the human anatomy. Female cynomolgus monkeys share with women comorbidities common to age- and hormone-related health problems, including heart disease, osteoporosis, breast/uterine cancer, and cognitive decline [18], and also share a well-defined menarche, premenopause (characterized by a 28-day menstrual cycle), perimenopause, and postmenopause [18]. The upright posture and pelvic location of the bladder and urethra, ultrastructure of the sphincter complex, and pelvic floor support are similar to those of women [17, 18].

Badra et al. developed and characterized a nonhuman primate model of ISD [12] and then assessed the long-term efficacy of autologous skeletal muscle precursor cell (skMPCs) on urinary sphincter structure and function [11]. Intrinsic urinary sphincter deficiency (ISD) was created by cauterizing and then transecting its pudendal innervation. The ISD procedure produced sustained (12-month duration) reductions in sphincter muscle content and reduced MUP. Local injection of skMPCs restored the muscle content and MUP values for 12 months, illustrating a model of stable ISD and treatment effects. Urodynamic studies were performed before and during pudendal nerve stimulation at baseline, and 3, 6, and 12 months after injury. Sphincter function was studied in vivo by cystourethrography, and ex vivo by quantitative histology and immunohistochemistry at these time points. The inflicted injury produced a 47–50 % decrease in maximal urethral pressure versus baseline [11, 12]. It also abolished the increase in maximal urethral pressure in response to pudendal and hypogastric nerve stimulation and this effect persisted for more than 12 months after injury. Urodynamic changes were consistent with decreased skeletal and smooth muscle content [11, 12], decreased nerve responses and an associated decrease in somatic and adrenergic innervation in the sphincter complex.

A translational weakness of the study of Badra et al. [11] was that the cells were injected only 6 weeks after muscle biopsy, which makes the approach more prophylactic than therapeutic, since a chronic, irreversible sphincter injury may not have been achieved. These studies, as well as a study by Cruz et al. [10], reporting beneficial effects of intravenous cell therapy on the lower urinary tract of rats with vaginal distension-induced pelvic floor deficiency, provided the rationale to perform a side-by-side acute (6-week duration) versus chronic (6-month duration) ISD; and urinary sphincter versus intravenous administration of cells in the monkey model of stable ISD. The present results support a reduced efficacy of cell therapy in chronic ISD, which is consistent with the results of clinical studies where cell therapy is only modestly effective [1, 2]. While intravenous administration of cells has been proposed for SUI and for other conditions [10], the results of this study do not show efficacy of this route of cell administration in chronic ISD. Only minimal effects were demonstrated in acute ISD.

The present study was not designed to explore the reasons why cell therapy is less effective in chronic ISD. However, the fact that reduced efficacy was associated with reduced innervation and vascularization, suggests that cells do not effectively stimulate the regenerative processes in chronic ISD. Whether this is a failure of cells to directly contribute to these processes, or through their indirect effects in stimulating cell mobilization or other paracrine effects, is unclear. It is known that cells secrete substances (secretomes) that stimulate regeneration of the pelvic floor in rats [19]. It has recently been reported that the chemokine CXCL12 stimulates urinary sphincter regeneration in a monkey model of chronic ISD, thus identifying a potential future therapeutic approach (cell mobilization) for women with chronic SUI [20].

A limitation of the present study is the lack of dose-response assessment of the number of cells needed to produce an optimal response. We only used a single administration of a fixed number of cells and we cannot exclude that a higher cell number or repeated injections could have produced better efficacy. Nevertheless, it is obvious that the number of cells that induces a marked improvement of sphincter structure and function when locally injected in acute ISD, has markedly reduced effects in chronic ISD. If applicable to the human situation, such reduced responsiveness may explain the often modest clinical effect of cell therapy in patients with ISD.

## Conclusions

The present results show that the route of administration influences the effect cell therapy has on urinary sphincter muscle structure and function, administration of a fixed amount of cells by local injection being superior to intravenous administration. Importantly, the effect of cellular treatment is reduced in chronic ISD, irrespective of route of administration, possibly related to the relative ability of cells to stimulate vascularization and re-innervation.

## Abbreviations

ALC: acute ISD/intrasphincteric (local) injection of vehicle without cells
ALM: acute ISD/intrasphincteric injection of skMPCs
ALV: local injection of vehicle only
ASM: acute ISD/intravenous (systemic) injection of skMPCs
CLM: chronic ISD/local injection of skMPCs
CLV: chronic ISD/local injection of vehicle
CSM: chronic ISD/intravenous injection of skMPCs
ISD: intrinsic urinary sphincter deficiency
MUP: maximal urethral pressure
NHP: nonhuman primates
skMPC: skeletal muscle precursor cells
SUI: stress urinary incontinence
